# Glances at the Hospitals

**Published:** 1901-09-21

**Authors:** 


### GLANCES AT THE HOSPITALS.

### THE BRISTOL ROYAL HOSPITAL FOR WOMEN AND CHILDREN.

The net ordinary income for the year 1899 was £3,657, and the ordinary expenditure was £4,422, showing a deficit of £765 on the work of the year. The year began with £2,811 on the wrong side; but special donations reduced this by £889, and £1,200 was transferred from the capital account, leaving an adverse balance of £1,487. The managers say it is clear that the income must be increased by £800 a year, or year by year some of the investments must be realised. It would seem, therefore, that the managers have no intention of reducing their expenditure as the only alternative. The workmen's contributions amounted to about £221, but doubtless more than this sum came from this class and was incorporated in the sums put down to friendly societies, &c. The patients' payments amounted to £404; and as 5,717 patients were under treatment during the year these payments amounted to only about 1s. 5d. per head. The cost of maintenance of the patients is not stated. At the beginning of the year the hospital contained 48 patients, and 688 new cases were admitted, making a total of 736 under treatment. Of these 505 were discharged cured, 69 improved, 26 not improved, 16 discharged for other reasons, 74 died, and 46 remained in the hospital. These numbers refer to children only. There were 4 women in residence on January 1st, and 45 were admitted during the year. Of these 49 cases, 35 were cured, 6 improved, and 4 not improved. The table inserted at page 30 shows the diseases from which the patients suffered, but does not give the results. Another table gives the number of deaths and the cause of death in each case. Among the children the percentage of deaths was 10·7. There is no table showing the year's surgical operations, nor, except in the dental department, is the anæsthetic in use named.

### THE BURTON-ON-TRENT GENERAL INFIRMARY.

The subscriptions amounted to £2,656, and the expenditure to £3,111. The investment account has been added to by £2,000 from the estate of the late Mrs. Worthington, and by a donation of £50 from the Bass Football Association. The hospital has been considerably added to, and now contains 72 beds. Neither the cost per bed occupied, nor the cost per patient, nor the cost per head per week is stated. The in-patients numbered 501 in addition to 35 in the building at the beginning of 1899. Of these 536, the recoveries numbered 348, the relieved 39, the not relieved 6, the deaths 42, and 51 remained under treatment. The average stay of each patient in the hospital was nearly 27 days. The out-patients numbered 1,304. The medical and surgical statistics are unusually meagre, and are compressed into a page and a half of the Report. One table names the surgical diseases, injuries, and medical diseases, and the number of patients who suffered from each disease; but no results are forthcoming. Another table gives the operations of the year and the results, and another gives the causes of death. There is no mention of the anæsthetics used.

### THE ROYAL HOSPITAL, BATH.

At the end of 1899 the bank overdraft was £1,324, and bills to the amount of £643 remained unpaid, so that the adverse balance amounted to £1,967. The Governors do not say in what manner they intend dealing with that deficit. The cost of maintenance is not stated. The in-patients numbered 1,289. Of these 1,067 were discharged recovered or relieved, 56 not improved, 85 died, and 81 remained in the hospital. The out-patients numbered 8,941. These figures show that during 1899 the in-patients were 99 in advance of the numbers for 1898, and that the out-patients were 732 in advance of 1898. As to the latter constant supervision on the part of the hospital authorities is essential to prevent abuse. The medical and surgical statistics convey much information. Each disease has columns showing the number of patients and the results of treatment; and this accuracy is carried out even in the table of cases visited at their own homes. It is not a little strange that the table of operations is not supplied with these columns, and we only get the numbers operated on in the various diseases or injuries. The anæsthetics table shows that nitrous oxide was given 199 times; A. C. E. mixture, 177 times; gas and ether, 170 times; chloroform, 142 times; chloroform and A. C. E., 48 times; ether, 17 times; gas, ether, and A. C. E., 8 times; gas, ether, and chloroform, 6 times; and chloroform and ether, 6 times.

### THE BOLTON INFIRMARY AND DISPENSARY.

The income for the year 1899 amounted to £6,610 and the expenditure to £6,077, leaving a balance on the right side of £533, a condition of financial affairs which must be as gratifying to the governors of the hospital as to the subscribers who entrust them with the management. The average cost per head does not appear in any part of the report so far as we could see. The in-patients numbered 1,184, the out-patients 3,133, the home-patients 554, and the casualties 2,356, making a total of 7,236 patients under treatment during the year. The daily average number of in-patients was 91, and the average residence of each was 29 days. In the eye department 59 in-patients and 567 out-patients were treated. The surgical operations numbered 515, and the mortality among the patients operated on was 4·4 per cent. There were 93 deaths in the hospital, being a percentage of 7·8. Of these deaths 36 occurred within 24 hours of admission, and if these be deducted the death rate would fall to 4·8 per cent. The medical and surgical tables are very carefully prepared. The diseased are arranged into groups, and the total number under treatment is placed in one column, the recoveries in another, the non-recoveries in a third, the numbers remaining in a fourth and a fifth one shows the deaths. Finally there is another column for "remarks." The surgical operations are detailed in the same careful way. We only miss a table showing the nature of the anæsthetic employed.

### THE SALISBURY INFIRMARY.

The receipts for the year ending July 1899, were £4,502, and the expenditure was £5,521. At the beginning of the year there was an adverse balance of £2,756 and at the end of the year it had increased to £3,618. It would seem from the report of the committee of management that there has been a general falling off in the annual subscriptions; and we notice from the abstract given on page 9 that the amount received under this head has gone down from £1,340 in 1890 to £1,120 in 1899. At the annual court proposals were to be made with a view of reducing the indebtedness of the hospital to the treasurer. The hospital contained 100 patients on July 30th, 1898, and 805 patients were admitted during the twelve months ending July 30th, 1899. The out-patients numbered 3,636. The medical and surgical tables convey no information beyond the numbers of patients treated for the various diseases; but the table of operations is well arranged and gives details in all cases. There were 277 operations, and in 15 of these death followed. An anæsthetic was used in 247 instances, but the particular anæsthetic used is not stated. There is also a table showing the cause of death in each of the 48 patients dying in the hospital.

### COVENTRY AND WARWICKSHIRE HOSPITAL.

The total ordinary income for the year ending October 31st, 1899, was £3,302. Although the item of annual subscriptions shows a slight increase over the previous year, there is a decrease in every other item of hospital income; and as the ordinary expenditure amounted to £3,523 there was a deficit of £221 on the year's working. It is therefore not without cause that the committee earnestly impress upon the subscribers and the public the urgent need for more liberal support. At the beginning of the year there were 53 patients in residence, and 653 were admitted during the 12 months, making a total of 706. Of these, 415 were discharged cured, 166 relieved, 9 not relieved, 1 at his own request, 45 died, and 70 remained under treatment. The out-patients numbered 5,406. The medical and surgical tables merely name the diseases and state the number of patients suffering therefrom.

### THE ROYAL HOSPITAL, RICHMOND, SURREY.

The total income for the year 1899 was £4,835; but of this sum £1,374 was derived from legacies. The annual subscriptions show a slight increase, while the donations are lower than in the previous year. The committee refer to the exertions of Dr. Cundell, whereby a sum of £3,000 was raised and has been invested for the perpetual endowment of the hospital. The ordinary expenditure amounted to £3,539. This left a sum of £1,000 to be transferred to the extension fund, and there was a balance of £293 in the treasurer's hands. The average cost per bed occupied was £73 6s. ; the average cost of each in-patient was £6; and the average cost per diem was 4s. There were 45 patients in the hospital at the beginning of the year, and 523 were admitted during the year, being a total of 568. The medical and surgical tables give the number of patients treated for various diseases, but no other information. A table shows the cause of death in the 43 patients who died during the year.

### DURHAM COUNTY HOSPITAL.

THE income for the year 1899 was £2,714, and there was a balance of £1,189 over from the previous year, which made a total of £3,903. The expenditure was £3,447. There were 45 patients in the hospital at the beginning of the year; 560 were admitted during the year, making a total of 605 under treatment. Of these 448 were cured, 95 relieved, 32 died, and 30 remained in the hospital on the date of report. The total number of out-patients was 2,240. Of these 2,079 were cured or relieved, 6 died, and 155 remained on the books; 468 patients were visited at their own homes. This is one of the hospitals whose report contains neither medical nor surgical statistics.

### ROYAL NATIONAL HOSPITAL FOR CONSUMPTION AT VENTNOR, ISLE OF WIGHT.

The total receipts for 1899 amounted to £21,547 ; but this included legacies for upwards of £10,000. The ordinary expenditure amounted to £13,254, and the extraordinary expenditure to £9,700, making a total of £22,954. It is pointed out that the splendid situation of this hospital in the sheltered undercliff affords the patients the very best chance of recovery by the open-air treatment, the principle of which was pointed out thirty years ago by Dr. Hassall, the founder of the hospital. Here the patients' bedroom windows are kept open at night and during the day, and the same principle is applied to the sitting-rooms as far as possible. The rooms are all ventilated on the "vacuum" method, which ensures the extraction of 5,000 cubic feet of air per hour per patient. The greatest care is taken to prevent the transmission of the germs of the disease. We learn from the medical report that during 1899 there were under treatment 880 patients. Of these 248 were very much improved, 345 were much improved, 123 were not relieved, and 13 died. The "very much improved" cases reached a percentage of 35·39, and this would be considered a high proportion in most consumption hospitals. It is rendered more remarkable from the fact that only 106 of the total number of patients were in the first stage of the disease, when it is, of course, much more amenable to treatment; 188 were in the second stage, and 415 were in the third or cavitation stage. The following new rule has been made by the committee: "The persons eligible for admission must be necessitous, and not in a position to defray the entire cost of maintenance and medical treatment, and not in receipt of parochial relief." The report is an interesting one, and in addition to the usual matter, contains pamphlets by the National Association for the Prevention of Consumption.

### WOLVERHAMPTON GENERAL HOSPITAL.

THE total receipts were £9,929, and the total expenditure was £9,913, so that there was a small balance at the end of the year's working; a satisfactory state of affairs, more especially as £114 was spent on ventilating the operation theatre and on electric fittings. The Hospital Saturday collections amounted to £4,250. The average daily number of in-patients was 131; the average cost per head was £3 2s. 2d., the cost per bed occupied was £53 2s. d., and the average stay in the hospital was 22½ days. The total number of in-patients during the year was 2,240. Of these 1,835 were discharged cured or relieved; incurable or discharged at own request, 100; died, 185; but of the latter 48 arrived at the hospital in a hopeless state. At the close of the year 120 remained under treatment. The number of out-patients treated during the year was 16,045. Of these 6,299 were medical, and 9,143 were surgical. As accidents, or urgent cases without tickets, 5,952 were treated. The average number of attendances per week was 1,156. The table of operations performed runs to 7 pages, and in every case the age and sex of patient, and the result of the operation are given. Eighteen amputations were performed and all recovered except one who died of phthisis. There were four cases of ovariotomy, all successful. A complete summary of the operations appears on page 115, from which it seems that 661 operations were performed, that 571 of these recovered, 42 relieved, 22 not relieved, and 26 died. An analytical table of diseases and injuries is given, and the results are clearly shown in every case. A short history of the hospital is given. It was opened in 1849, for 80 beds, and cost £18,898. It has been altered and added to at various times, and now the total cost has exceeded £44,000. The report is a very well arranged one. We only miss a table of the anæsthetics used in the hospital.

### THE BLACKBURN AND EAST LANCASHIRE INFIRMARY.

THE total receipts from all sources amounted to £7,321, being a decrease of £150 on the previous year. £1,250 was transferred to the building fund, and a balance of £681 was left to commence the following year with. There were 1,370 in-patients during the year. The average stay in the hospital was 26⅗ days, and the average daily number in residence was 83. There were 82 deaths, but 25 of these took place within 48 hours of admission, and if these be omitted the average death-rate would be 5 per cent. 4,275 patients were attended to in the out-patients' department. 329 operations were performed during the year, and the tables show the results of these, and also in the medical and surgical cases throughout. We failed to notice any table showing the anæsthetic used.

